# Hierarchical Nanobiosensors at the End of the SARS-CoV-2 Pandemic

**DOI:** 10.3390/bios14020108

**Published:** 2024-02-18

**Authors:** Jael Abigail Medrano-Lopez, Isaela Villalpando, Ma Isabel Salazar, Carlos Torres-Torres

**Affiliations:** 1 Sección de Estudios de Posgrado e Investigación, Escuela Superior de Ingeniería y Eléctrica, Unidad Zacatenco, Instituto Politécnico Nacional, Mexico City 07738, Mexico; 2 Centro de Investigación para los Recursos Naturales, Salaices 33941, Mexico; 3 Departamento de Microbiología, Escuela Nacional de Ciencias Biológicas, Instituto Politécnico Nacional, Mexico City 11340, Mexico

**Keywords:** SARS-CoV-2, hierarchical nanostructures, nanoparticles, biosensors, detection limit

## Abstract

Nanostructures have played a key role in the development of different techniques to attack severe acute respiratory syndrome coronavirus 2 (SARS-CoV-2). Some applications include masks, vaccines, and biosensors. The latter are of great interest for detecting diseases since some of their features allowed us to find specific markers in secretion samples such as saliva, blood, and even tears. Herein, we highlight how hierarchical nanoparticles integrated into two or more low-dimensional materials present outstanding advantages that are attractive for photonic biosensing using their nanoscale functions. The potential of nanohybrids with their superlative mechanical characteristics together with their optical and optoelectronic properties is discussed. The progress in the scientific research focused on using nanoparticles for biosensing a variety of viruses has become a medical milestone in recent years, and has laid the groundwork for future disease treatments. This perspective analyzes the crucial information about the use of hierarchical nanostructures in biosensing for the prevention, treatment, and mitigation of SARS-CoV-2 effects.

## 1. Introduction

Sensors based on hierarchical nanostructures in the area of nanomedicine have been meticulously investigated in order to identify different enzymes and organisms such as bacteria or viruses. Biosensors are fascinating instruments that basically serve to detect biological or chemical parameters such as those related to molecules in tissues, microorganism cultures, and nucleic or acid chains [1]. The characteristics related to biodetection like selectivity, response speed, and stability depend on the morphology and structure of the sensing materials [2].

The main types of sensors used in biodetection are electrochemical [3], thermometric [4], piezoelectric [5], magnetic [6], and optical sensors (plasmonic [7], UV-Vis/infrared spectroscopy [8], Raman and SERS [9], or attenuated total reflection [10]). Biosensors that are developed using hierarchical nanostructures can be manufactured with different nanomaterials. For example, nanohybrids can be integrated into diverse materials such as noble metals [11], graphene [12], copper, titanium [13], zinc oxide [14], and bimetallic oxide [15], among others. The biosensors can be classified into three groups according to their mechanisms: the biocatalytic group that uses enzymes, bioaffinity group that involves antibodies and nucleic acids, and microorganism group that uses microbes [16].

A strong selective control of the manufacturing parameters of noble metals is possible [17], allowing their structure to be modified [18] to improve their physicochemical properties and adjust their shape [19]. There are multiple techniques for designing nanobiosensors, but the most common ones are based on electrochemical deposition [20], electroless deposition [21], electrocatalysts [22], and physicochemical methods [23].

Besides different processing routes that have been extensively explored to improve biosensing effects, the use of the LSPR phenomenon is very attractive [24], and the development of hierarchical nanostructured biosensors can promote exceptional optical, electrical, and chemical properties based on LSPR. Some of the special characteristics exhibited by hierarchical nanostructures are derived from their ultra-high specific surface area, high flexibility, light weight, high electrical conductivity, and bio-compatibility [25,26,27,28,29,30].

The hierarchical nanostructures are replacing conventional random hybrids in counterparts thanks to their physical characteristics, stability, and efficient transfer of electronic and ionic charges [31,32]. For example, their morphologies show a high surface area with adjustable porosity or packing density. Some hierarchical assemblies serve as programmable scaffolds that provide molecule-level control over the distribution of fluorophores and nanometer-scale control over their distance. Several strategies can be used to study imperfections and to stabilize various types of nanostructures, such as hollow ones [33] or cage frames to obtain a better performance [34].

It is worth noting that hierarchical metamaterials have been reported for the development of virus-based light learning systems, in plasmonic structures for application in high-performance metamaterials, and in binary nanoparticle networks and liquid crystal arrays for sensing technologies and imaging [35]. With these procedures, diverse techniques have been demonstrated strong fluorescence intensity and mild levels of enhancement, which allows them to manipulate photonic excitation and photoemission [36].

Hierarchical nanostructures represent a potential key to the next generation of new nanomaterials. For example, a controlled structure in the agglomeration between nanoparticles can increase plasmonic effects while the stacking distance between other nanoparticles decreases; all of this can be used to develop new and effective detection methods. Some of the representative hierarchically structured shapes are nanopillars [37], nanocones [38], nanoholes [39], and gecko pillars [40], among others.

Hierarchical nanostructures can be fabricated using techniques such as nanosphere lithography [41] with multiple patterns [42], electron beam lithography [43], pattern transfer [44], and focused ionization [45].

The characterization of the morphology, structure, and stability of hierarchical nanostructures can be explored by different methods. The typical characterization techniques for hierarchical nanostructures are X-ray diffraction [46], electrical effects [47], TEM [48], energy dispersive spectroscopy (EDX) [49], AFM [50], optical interactions [51], PL [52], Brunauer–Emmett–Teller surface area analysis [53], UV–visible absorption spectroscopy [54], photovoltaic performance [55], photocatalytic processes [56], Raman spectroscopy [57], and magnetic phenomena [58].

A hierarchy in nanostructures can be developed through in situ plasmon-driven syntheses [59] or through amino acids [60] to easily detect analytes at trace levels, such as pesticides, heavy metals, explosives, proteins, pathogens, and other chemical and biological contaminants [61]. It is clear that nanomaterial sciences are essential for developing biosensors with high reliability and speed using innovative technology [62,63,64,65].

In the last two years, diverse experiments have been carried out in the development of biosensors using different hierarchical nanostructures. It is worth highlighting some examples that have been very useful in the commitment to developing biosensors with better properties.

It has been pointed out that biosensors can be used to see the effectiveness of the vaccines in healthy, convalescent, or vaccinated people [66]. They can be used to monitor diseases, observe how many antibodies exist in people’s fluids, as well as determine whether the vaccines are effective for the test subjects [67]. In the faster biosensors, it takes approximately 20 min to obtain the result. The research has sought to develop biosensors with these nanomaterials to achieve a relatively rapid response, achieving a response time of 15 min.

It has been observed that current biosensors also have some disadvantages such as not being capable of detecting analytes in samples when there are external stimuli. This has to be addressed with the development of different biosensors with the properties of nanomaterials, such as different probes, including plasmonic [68] and incorporated ones [69]. Biosensors capable of detecting pathogens with very little genetic material compared to other assays have also been developed [70]. Additionally, calorimetric strips for smartphones aimed at antibodies or antigens to combat the rapid spread of these diseases have been considered since wearable biosensors can constantly monitor patients [71].

**Figure 1:**
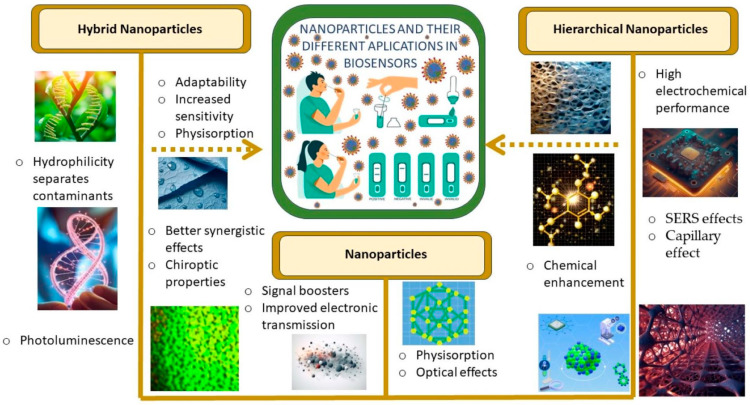
Representative characteristics exhibited by different nanostructures in biosensing applications.

With this motivation, this paper reviews different aspects of the cutting-edge biosensors in the detection of SARS-CoV-2, focusing in those based on hierarchical and hybrid nanoparticles. Figure 1 shows the main characteristics considered in our research.

## 2. Synthesis of Hierarchical Nanostructures and Multicomponent Assemblies for Biosensors

Materials with hierarchical nanostructures have excellent mechanical properties due to the functional adaptation of their structures into different hierarchical levels. Hierarchical structures can be observed in nature, such as in bones, wood, cork, and plant stems, or in glass sponges [72]. Hierarchical nanomaterials show different architectural designs that are ordered at multiple length scales. They are grouped according to their main characteristics; in the case of porous materials, they contain interconnected pores with at least two levels of pore hierarchy from molecular (1–100 A), nano (10–100 nm), and meso (1–100 μm), to macropores [73]. It should be noted that the construction of hierarchical nanostructures requires knowledge of particular principles to avoid limitations on their properties [74]. Hierarchical materials can mimic the mechanical properties of their biological counterparts. Smart hierarchical materials can exhibit specific stimulus-response properties [75], such as self-healing and self-regeneration [76] in order to improve fracture resistance and increase strength [77]. Arrays can be constructed using proteins and microscale mechanical constraints can be used to form ordered networks within macroscopic structures [78]. The synthesis at different orders of magnitude from nanoscale to macroscale can be used to acquire outstanding characteristics through interacting with different analytes of different sizes [79], from small proteins to living cells. Different networks can be designed according to the geometry of the templates used [80]. The nanoclusters can be protected by ligands that can be prepared with atomic precision, exhibiting well-defined structures and resulting in versatile building blocks to manufacture excellent structures capable of performing certain functions [81]. For instance, nanofibers are used to construct multifunctional walkways with up to five levels of organization (depending on the method used). In the first level, there is a composite nanofiber; in the second level, a layer of composite material coated on the composite nanofiber that will result in the third level. The fourth level organizes the nanofibers to form an assembly and finally, in the last level, an assembly of nanofibers can be encapsulated within a matrix to form a massive structure by default [82]. Nanotubes are commonly used for the manufacture of hierarchical materials since they consist of molecular blocks, whose characteristics can be related to an anisotropic supramolecular self-assembly behavior at a personalized nanoscale, which allows for the creation of a percolation network at the mesoscale. They are regulated by dynamic self-assembly into four hierarchical levels of self-organization [83]. Nanosheets are composed of 2D building blocks, which have atomic or molecular thicknesses and they are considered the thinnest functional nanomaterials. They can be organized into various nanostructures or combined with a variety of materials at the nanoscale. Thanks to this, wide-range assemblies such as organic molecules, polymer gels, and inorganic nanoparticles can be designed [84].

Although hierarchical nanomaterials can be considered hybrid materials [85], nanohybrids are composed in a different way. Hybrid materials can have a variety of complex architectures with or without hierarchy. Their size varies from nanometers to several micrometers and several millimeters. Hybrid nanomaterials are combined through the synergistic mixture of two or more nanomaterials, which can be either inorganic or organic [86], that create a single material with properties that go beyond their properties as individual elements. They consist of groups of blocks with similar properties and structures with groups that cross-link the polymer into chains [87]. Their properties are determined by a combination of structure and composition at each length scale [88]. As a result, their properties are expressed in molecular length scale structures [89]. This indicates that the new mixture has superior properties compared to the original mixture. The properties to look at are the advantages derived from nanomaterials at a macroscopic level, such as energy absorption performance. The lightweight structure maximizes its functionality and improves the efficiency of the material [90,91].

There are different forms of nanohybrids such as sandwich structures, foams, reticular structures, segmented structures, zero expansion [92], and meso-structured thin films [93]. These structures serve different purposes, especially in integrated refractive and diffractive optical devices. Since these nanomaterials have a large thermal stability and better compatibility, they are typically used in the production of semiconductive devices [94]. In order to characterize organic–inorganic materials, techniques such as FTIR, Raman spectroscopy, LSPR, and various techniques based on MS are used [95].

Hybrid nanomaterials are good candidates for developing nanomaterials in the fight against bacteria and viruses thanks to their high sensitivity, good stability, and selectivity. In particular, they can detect antigens in plasma since their good electrochemical activity helps in the immobilization of the chains of different aptamers [96].

Nanomedicine, based on hybrid and hierarchical nanomaterials, has achieved great progress in the field of biosensors for the diagnosis, prevention, detection [97], and treatment of diseases in the post-pandemic period [98]. Compared to bulk materials, nanostructures are more precise, more reliable, less invasive, and easier to carry according to their chemical elements [99]. The effectiveness of nanomaterials has advanced to detect diseases at a very early stage using new technologies based on nanobiosensors [100], whose physical principles at the nanoscale level allow the biological receptors to be highly sensitive [101]. Nanobiosensors can be tailored by using different types of nanomaterials and structures [102].

Depending on their interactions, nanobiosensors can be classified into two different groups called biocatalytic or biophilic. These two groups can be classified according to recognition factors, for example, cells, organelles, tissues, enzymes, receptors, antibodies, nucleic acids, MIPs, PNAs, or aptamers.

Nanostructures are capable of obtaining information through molecular interactions in real time, and in normal and pathological biological states which provides an effective and relatively fast result. For example, in a drop of blood, an enzyme such as glucose oxidase, glucose dehydrogenase, or hexokinase can cause a reaction, which can be measure by a low-dimensional detector in a glucometer (biosensor) [103].

Because the manufacturing of biosensors has several drawbacks, efforts have been made to develop improvements in manufacturing [104,105,106,107,108]. Characteristics like adhesion ability, strong adsorption capacity, chemical catalytic efficiency, and corrosion and oxidation resistance facilitate the fabrication [109], chemical stability, and electron transfer kinetics [110]. The challenges for optimizing highly selectivity binding properties are continuously being overcome to analyze nanoscale elements of biomolecules [111].

High crystallinity with insignificant structural defects can be relevant to detecting different samples such as glucose, proteins, and nucleic acids [112]. The other main advantages of nanohybrid materials are the specific binding sites that generate a selective sensor signal, which also improves its magnitude and composition. The high surface-to-volume ratio of nanofibers can also improve the capture efficiency and it provides some surface area-related phenomena, including ion exchange and catalysis [113,114].

Heterounions in hierarchical nanomaterials can promote the selective formation of specialized structures and sensitive responses not found in other sensors [115,116,117,118]. Nanomaterials produced through molecular printing can create selectivity for specific enzymes. This method can be worked with 3D nanostructures and it is used to manufacture versatile materials for the construction of sensors to detect various analytes [119]. It has been demonstrated that these nanostructures can eliminate pathogens and better detect enzymes compared to other nanomaterials [120].

The existing improvements found when assembling nanostructures are versatile, and they open new methods for different technologies to control their structure and combine physicochemical properties [121,122,123,124,125,126,127,128,129,130,131]. On the other hand, some nanostructured systems based on organic polymers have been proposed [132], and applications for spectrochemical biosensing have been demonstrated. Biosensors based on RNA hybridization can be considered for several biological reactions and for generating analytical signals that are easily detected by different electrochemical aptasensors [133], electrochemical luminescence sensors [134], and optical transducers, among others [135]. It has been pointed out that RT-PCR [136] can be used to amplify cDNA from virus RNA [137]. This is of great interest in the studies that have to do with inhibitors that target the enzyme helicase since it is known to participate in the processes of duplication and cell reproduction [138].

Photonic nanobiosensors have been also highlighted with respect to their potential use against SARS-CoV-2. In addition to monoclonal antibody pairs, which are rapid antigen tests [139], it is important to look for more efficient ways to detect pathogens [140]. In this direction, biosensors using some promising plasmonic nanoparticles are the most powerful tools employed for the detection of viruses [141]. Moreover, polystyrene nanoparticles, graphene, and carbon nanotubes [142] present different advantages such as selectivity towards particular molecular expressions corresponding to an important challenge that requires high specificity, sensitivity, and a multiplex detection capability to offer good virus detection. The design of POC testing arrangements [143], such as LFIAs, should be mentioned as they offer fast and easy-to-use methods, as well as reliability. Each synthesis procedure can be functional, but inherent limitations in the quantitative analysis of the virus in the application of biosensors should be noted [144].

**Figure 2:**
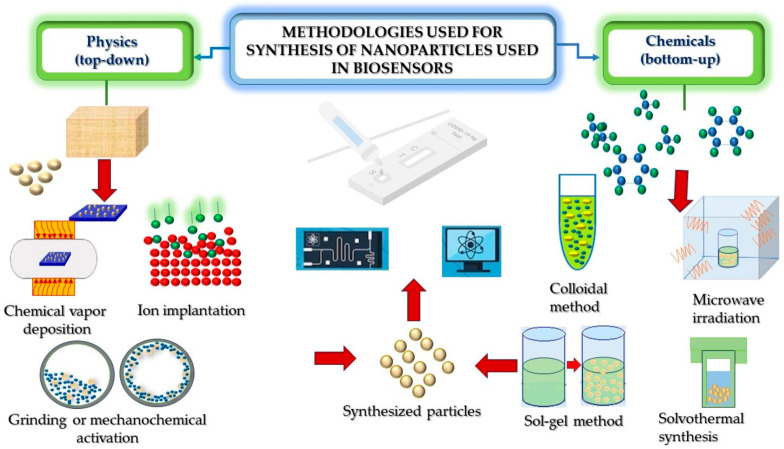
Representative processing routes for the synthesis of hierarchical nanostructures used in the development of biosensors.

In summary, hierarchical nanostructures are formed by hybrid nanoparticles, which are good candidates for the development of nanobiosensors for pathogen detection. These nanoparticles are prepared through different synthesis methods, as it is illustrated in Figure 2.

Hierarchical and hybrid nanostructures allow for the improvement of biosensing performance by increasing the signal intensity and enhancing a variety of energy transfer processes. Hierarchical nanostructures have larger reaction interfaces in the specific active surface area, allowing for better biomolecular recognition, catalyst charge transfer, metal ion release, and virus or bacteria capture within the nanomaterials. Sensors developed with these nanomaterials have high sensitivity, as shown in Table 1.

## 3. Hierarchical Nanostructures for SARS-CoV-2 Biosensing

In particular, biosensors made from nanostructures have a good response speed, good mechanical and chemical stability, a simple manufacturing process, low cost, as well as selection of samples in situ [162]. In order to develop nanobiosensors for detecting an infection, hierarchical nanostructures can be employed to assist in the synergistic enhancement of molecular enrichment [163]. An example is polydimethylsiloxane (PDMS), a transparent and flexible substrate that can be wrapped on arbitrary surfaces and allows light to penetrate the contact surface for optical diagnosis in situ [164]. Optical and plasmonic biosensors have been investigated with different nanostructures to combat diverse types of viruses, such as those that imitate different viruses called “virus traps” and elongated nanoparticles [165,166,167]. Biosensors based on electrochemical detection have been developed for identifying different types of viruses such as nanobioconjugated nanostructures. Virus coat protein self-assemblies with nanostructures can be very symmetrical [168]; a good example is the gold-functionalized nanostructures with human ACE2 that have a detection limit below approximately 80 mL^−1^ copies. Another example is the nanostructured biosensing process in contaminated water, in which, the contaminant can be detected in a few minutes. Hierarchical assemblies are very promising in combating not only the SARS-CoV-2 virus, but also different pathogens. The evolution of the self-assembly pattern of the nanocomposite can alter their plasmonic response and can be used for molecular diagnoses [169]. Table 2 compares the advantages and disadvantages of representative characteristics exhibited by hierarchical nanobiosensors.

Hierarchical nanomaterials in detection platforms can be tailored for the construction of functional electrode nanomaterials [186]. The aim of hierarchy in biosensing is to form nanomaterials that are biomolecular self-assemblies for the enhancement of sensing [187]. Some hierarchical structures are inspired by nature since these nanostructures are functional [188] and because they contain properties that make them unique. As it can be seen in Table 2, hierarchical nanostructures are attractive for applications in the detection of pathogens. For example, biosensors using hierarchical metal nanoparticles that are monitored with SERS techniques are capable of accurately measuring and capturing unknown coronaviruses, as long as they contain the S protein and can combine with the ACE2 protein [189]. There is efficiency in the transfer of resonance energy between nanomaterials made with noble metals. These nanomaterials exhibit enhanced electrochemical conductivity when combined with hierarchical materials such as graphene, resulting in pronounced absorption peaks in the hybrid materials. For example, their narrow pore size distribution results in faster direct electron transfer and better enzyme detection [190]. Based on this, enzymatic biosensor platforms have been created, as well as highly conductive supports and nanofillers, which demonstrated the effectiveness of hierarchical nanostructures [191]. This approach results in a linear and continuous reaction that increases the uptake of RNA from the viral material. The hierarchical nanostructures of certain hybrid nanomaterials enhance their synergistic effects [192], generate hydrophobicity and colloidal stability, and can be reprogrammed to detect any protein antigen if the corresponding specific monobody exists. They can increase colorimetric fluorescence signals [193], ensuring high sensitivity and stability for detection applications. Different nanomaterials can be used to detect metals and compounds such as copper [194], nickel oxide [195], etc. The modified assemblies are good candidates for chemical adsorption, and can produce good magnetization that generates a magnetic signal to detect pathogens such as SARS-CoV-2. The hierarchical nanostructures produce molecular enrichment through the capillary effect, resulting in good biocompatibility, stability, optical effects, and promising electrical properties.

**Figure 3:**
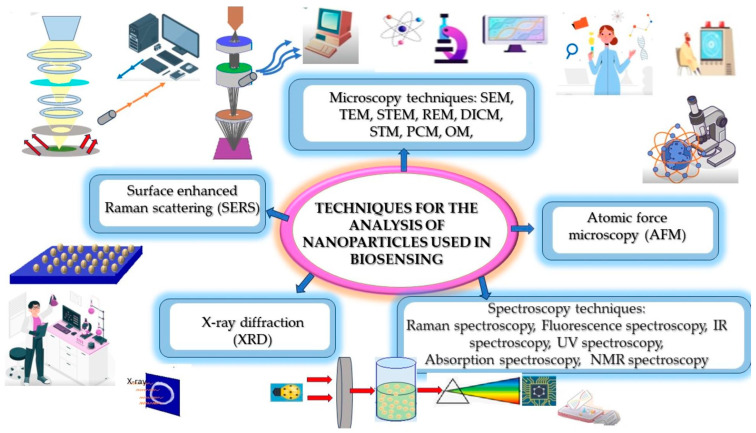
Typical techniques for measurements using nanostructured biosensors.

The characteristics of hierarchical nanostructures can be observed through different techniques, as illustrated in Figure 3.

## 4. Heterostructures with Different Morphologies for Biosensing

A fundamental element of nanoscience and nanotechnology are structure and morphology [196]. This is because nanostructured materials can be agonists due to their different shapes and the properties they acquire depending on their orientation. In this section, different types of nanostructures and nanomaterials are analyzed, such as nanowires, nanofibers, nanotubes, quantum dots, nanosheets, nanocomposites, and nanoparticles, and their different orientations, morphologies, and properties are discussed. Nanostructures come in a variety of shapes, sizes, structures, and origins. They can be spherical, conical, helical, cylindrical, tubular, flat, hollow, or irregular in shape. Their structures vary in size from 1 to 100 nm and are composed of elements such as carbon, metals, metal oxides, and organic or inorganic materials.

The development of biosensors using nanoparticles has become extremely important because nanostructured nanoparticles have properties that surpass other compounds such as a low detection limit, high sensitivity, biocompatibility, and a peculiar refractive index change. By being able to combine different materials to form heterostructures, it is possible to overcome the drawbacks of the individual materials and thus take advantage of their synergy [197].

The development of hierarchical nanostructures has become crucial for the tailoring of semiconductor-based nanostructures in the field of photoelectrochemical biosensing [198]. The sensors with enhanced biosensing performance include electrochemical, surface plasma resonance, photonic, immunosensors, photoelectrochemical, nucleic acid-based and enzyme-based, electrochemiluminescent, protein-based, and aptamer biosensors. Their adaptability in terms of size, shape, and composition is relevant in controlling their physicochemical properties [199] and general behavior to adapt nanostructures to biological systems [200]. Modifications of heterostructured effects such as the degree of exfoliation, crystallinity, phase, metallicity, sheet size, and decoration with metallic particles can be useful for biosensing [201]. Moreover, the arrangement of semiconductor nanostructures in biomolecules can convert this adaptation into hybrid systems to improve detection [202].

There is a goal to fabricate biosensors at a low cost in the fight against highly infectious diseases [203]. Different nanostructures have been proposed for designing multifunctional effects and nano-sculpted hybrids [204].

**Figure 4:**
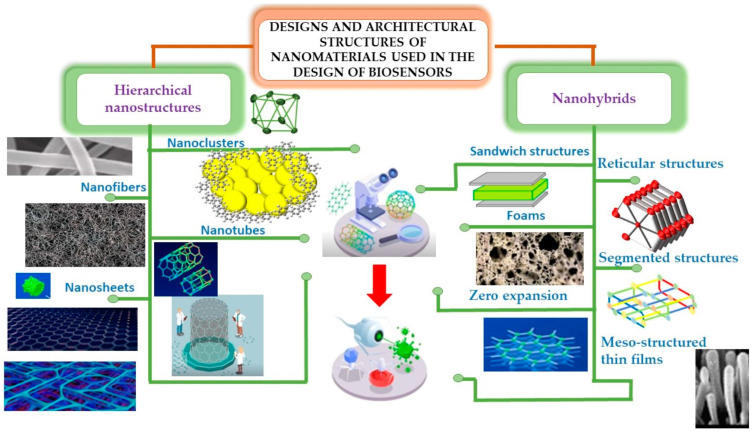
Representative characteristics exhibited by different types of nanostructures.

In this section, we will analyze the different types of heterostructures for biosensing, shown in Figure 4, which shows the particular morphologies of both hybrid materials and hierarchical structures.

Multidirectional networks of nanoobjects like nanocrystals or nanoflowers can im-prove their functional characteristics, which are defined by the synergy of different elements. The synthesis of heterostructures can emerge from the sequential growth of different nanoparticles or by the sequential preparation of different self-assembled building blocks. Morphologies like core–shell, multiple-shell, forest, nanoflowers, nanograss, nanopyramids, janus, and branched-tree heterostructures are typical nanostructured architectures. Table 3 presents some advantages and disadvantages of different morphologies of hierarchical nanostructured forms. The parameters related to the shape, size, distribution, and homogeneity of the building blocks are highlighted.

## 5. Discussion

The 2020 pandemic killed millions of people around the world, increasing the need to find more effective ways to combat the disease. This has served to curb deaths as new knowledge about the virus has been quickly found, opening new windows into the age of nanotechnology. During this pandemic, various applications have emerged to combat the risks caused by SARS-CoV-2. With the aim of developing various methods for the prevention, detection, treatment, and diagnosis of diseases, diverse solutions including vaccines, drugs, and biosensors have been engineered. One of these effective methods is the use of biosensors designed with hierarchical nanostructures that have unique properties to combat COVID-19, offering reliable, effective, and fast solutions [224]. Currently, researchers are looking for ways to produce materials with a low cost, high production volume, efficient cultivation times, maximum durability, and profitability. Good candidates for meeting these objectives are hierarchical nanostructures, as they are selective and they reduce the time of detection of potentially infectious pathogens. The advantages of developing these hierarchical nanostructures are that they can develop double resonance effects or synergistic effects of the elements that are integrated into the system [225] and have a high potential for advanced photonic monitoring [226]. A previous study mentioned several options for preparing hierarchical nanostructures, since each preparation has a specific purpose [227].

The preparation of these nanostructures is through different synthetic and analytical routes, which results in remarkable applications [228]. The controlled synthesis of hierarchical nanostructures with stacked morphologies could serve as a basis for tuning effects and potentially controlling them using photonic signals [229]. By designing complexes with multiple hierarchical levels [230], different nanomaterials can be developed. These complexes are envisioned with nanohybrid nanomaterials since their recent use in biosensors has facilitated the exploration of different techniques to produce them, including recycled materials [231]. By joining nanoparticles, different properties can be obtained to obtain good biocompatibility and high covalent bio-bonds with specific proteins [232].

In addition to amplifying the detection signal, hierarchical nanostructures can be employed to label a specific sequence of a target during detection [233]. Some of these nanomaterials feature combinable virus recognition targets such as bio-AuNP hybrid nanostructures with metal oxide and carbon nanotubes [234], which have been combined by pathogen DNA hybridization [235]. These nanohybrids have improved oxidation catalytic activity, stronger signals, better colorimetry for virus detection [236], a wider linear range, a lower LOD, higher sensitivity, and a better response time [237]. All these properties significantly contribute to new rapid and cost-effective methods and have provided smart nanomaterials with great advantages in biosensor fabrication [238].

An example of hybrid nanomaterials is carbon-based biosensors that provide a good effectiveness when the active detection point has a high surface-to-volume ratio, which increases the probability of adsorption to a specific molecule [239]. Electrochemical biosensors mainly use porous carbonaceous materials, since the morphology of these biosensors, especially the pore volume and surface area, influences the electrochemical and catalytic activities. All of these properties are widely used for pathogen detection and surveillance. Biosensors with layered functionality have been developed for the detection of SARS-CoV-2. The methods used to improve performance are activation, doping, and dispersion of metal nanoparticles for the detection of various analytes, such as biomolecules, metal ions, contaminants, and food additives.

In addition to some analytical techniques to study the biosensing performance, optical interactions of hierarchical nanostructures can also be used to combat SARS-CoV-2 and other viruses, resulting in higher sensitivity [240]. This can be seen when nanostructures are used in the development of nanotechnological therapies, such as the development of antiviral drugs and nanoarchitecture-based vaccines assisted by biosensing [241]. An example of nanoparticles that contain these properties are those whose surfaces are coated with ionic nanoparticles. Metallic nanoparticles are used, which act as antiviral agents against RNA viruses because most recurrent respiratory diseases are RNA viruses that take advantage of the link between the virus and the host cell to enter the host cell [242]. From this, various functionalization and rapid viral immunodiagnosis strategies can be used [243].

The development of several hierarchical microchips with good results has been demonstrated; these microchips have a high sensitivity due to a primed surface for covalent immobilization of primary antibodies and ensuring strong binding between substrates [244].

**Figure 5:**
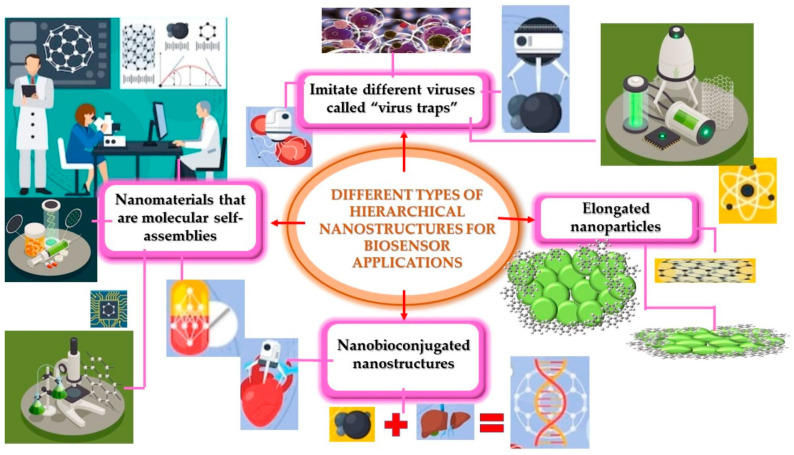
Different types of hierarchical structures for the development of biosensors.

Currently, the pharmaceutical profile of antiviral drugs has been improved and nanomaterials have been improved in terms of their virucidal properties and effectiveness against viral infections [245]. Figure 5 presents these different types of hierarchical nanostructures and how they are used for the development of nanobiosensors.

## 6. Conclusions

The development of hierarchical nanostructures is remarkable in the fight against SARS-CoV-2. In this paper, we reviewed different considerations for the development of hybrid nanostructures and their applications in biosensing. Nanomaterials with specific properties, such as noble metals, have become very useful for the development of vaccines and biomarkers thanks to their unique LSPR phenomena. In our analysis, we highlighted the advantages and disadvantages of the synergistic effects of the different elements integrated into nanoscale biosensors. The plasmonic properties exhibited by metallic nanoparticles together with the electronic or optoelectronic functions of carbon-based low-dimensional systems are good candidates for the advancement of biosensing technology. The use of hybrid nanomaterials for the development of biosensors for virus detection have resulted in great advantages, such as adaptability. In view of all these considerations we highlighted for hierarchical biosensing, we could obtain better results to combat the effects of some diseases using hierarchical biosensing, as was the case for COVID-19.

## Figures and Tables

**Table 1:** Advantages and disadvantages of hybrid nanomaterials, as well as their LOD for SARS-CoV-2 biosensing, focusing on the synthesis method employed for the preparation of the nanostructures.

Nanostructure(s)	Synthesis Method	Analyte(s)	Detection Limit	Advantages	Disadvantages	Reference
PPy and AuNPs	Electrochemical synthesis	DNA molecules from SARS-CoV-2	258.01 copies μL^−1^	Rough electrodes were found to have high electroactive areas that improve sensitivity and increase the number of active sites capable of recognizing target molecules	The sensing layer has an overall negative charge. This makes it difficult for electrons to pass between the transducer and the electroactive species	[145]
DNA tetrahedron with Au-gC_3_ N_4_ and PEI-Ru@Ti_3_ C_2_ @AuNPs	Electrochemical synthesis	SARS-CoV-2 RdRp gene	7.8 aM	The signal intensity increases, therefore the emission peak increases, therefore it effectively detects the RdRp gene of SARS-CoV-2	Two feet without the participation of bandage DNA cannot walk on the track because they are farther apart	[146]
Thiol-functionalized DNA aptamer flexible, carbon cloth coated with AuNPs	Electrochemical synthesis	SARS-CoV-2 spike protein	0.11 ng/mL and 37.8 ng/mL 0.167 ng/mL and 46.2 ng/mL	The proposed platform showed good mechanical stability, revealing negligible changes in voltammetric responses to bending at various angles. The sensor showed good selectivity and repeatability	Although the response of the AuNPs is adequate, upon binding with the Fe(CN)_6_ redox probe and by applying the transport characteristics to the mass of the electrode surface, the substrate can bend, which causes flexibility and could affect the voltammetric responses	[147]
Sandwich biotinylated signaling DNA–RNA hybrid nanobioconjugate with magnetic Au beads	Electrochemical synthesis	RNA from SARS-CoV-2 cell culture	807 fM	The genosensor demonstrated good sensitivity and a low detection limit. When the streptavidin poly-HRP 20 enzyme complex was used, it specifically detected SARS-CoV-2 and discriminated against homologous viruses in enriched samples and SARS samples	The electrochemical signal for the positive sample extracted from the cell culture had a lower electrochemical response because the genetic material (1.89 pM RNA/total DNA) was lower than the concentration of synthetic DNA tested (10, 100, and 1000 pM). Furthermore, the synthetic DNA samples correspond to a short ssDNA sequence, compared to the real samples that correspond to the entire SARS-CoV-2 genome	[148]
Au@Fe_3_O_4_ hybrid nanocomposites	Electrochemical synthesis	RNA of SARS-CoV-2	200 copies/mL	The resistance of the modified nanocomposite decreased drastically due to the high conductivity of AuNPs and RGO, which causes high sensitivity due to the good conductivity of these materials	The study was based on samples from the lower respiratory tract. However, upper respiratory tract samples are widely recommended for diagnosis because lower respiratory tract samples, especially for bronchoalveolar fluid and tracheal aspirates, have a high risk of aerosol generation	[149]
BPV with AuNPs	Ionic adsorption	SARS-CoV-2 via a self-assembled plasmonic nanoprobe array on spike proteins	1.4 × 10^1^ pfu/mL	The BPV enables strong NIR extinction peaks due to plasmonic nanogaps	In some studies, it has been shown that in adenoviruses, plasma has an effect dependent on the type of virus and, surprisingly, infectivity could be amplified for some types of adenoviruses. It is important to verify this information for security reasons if you plan to use it on other types of viruses	[150]
CaZnO-based nanoghosts	Radiochemical reduction	ssDNA, pCRISPR, and recombinant SARS-CoV-2 spike	10 nM	One of the key advantages of these NGs is their contactless interaction with cells/tissues/organs, which is a considerable advantage compared to other types of inorganic nanomaterials, including metal–organic frameworks (MOFs)	When performing this experiment, the fluorescence emission spectra did not show a significant decrease (presumably due to the lack of O-P bonds on the pCRISPR surface). The surface morphology of the completely quenched NG porphyrin showed little tubular structure for ssDNA	[151]
Bifunctional Au@Pt/Au nanoparticles	The Turkevich’s method (colloidal method) and galvanic replacement reaction	N protein of the SARS-CoV-2	1.27 pg/mL	Resonance energy transfer efficiency between Au@Pt/AuNPs	The protein shows some areas with agglomerates of particles, which can cause breakage in these agglomerates	[152]
DNA biosensor with AuNPs and graphene oxide	Modified Hummer method and Wang’s method	Targets four different regions of the SARS-CoV-2 viral genome	0.16 ng/μL	The GO/AuNP hybrid provides two significant absorption peaks, so the linear response and continuous increase in absorbance confirm the reliable and constant response of the biosensor towards the viral RNA	The first layer has the possibility of self-agglomeration of the AuNPs in the absence of RNA	[153]
Ag nanoparticles with ultrathin Au layers embedded with 4-mercaptobenzoic acid	Manufactured by a ligand-assisted epitaxial growth method	SARS-CoV-2 spike protein	0.22 pg/mL	Chemical advantages due to the surface of Au but also superior optical characteristics from Ag. It has great potential in practical clinical applications and can be used to detect and track the early immune response to SARS-CoV-2	Serum samples were used which increases the time for the analytical process	[154]
PEI with Fe_3_O_4_ core with dual layers of quantum dots	Electrostatic adsorption and chemical substance	SARS-CoV-2 nucleocapsid protein (NP)	0.235 ng mL^−1^ and 0.012 ng mL^−1^	One strategy of layer-by-layer modified assembly is that it involves electrostatic and chemical adsorption. Good magnetization which can provide magnetic signals for SARS-CoV-2 NP detection	The SM values of the materials gradually decreased because the proportion of the Fe_3_O_4_ core gradually decreased	[155]
MagTQD nanocomposites with Fe_3_O_4_	Colloidal method	SARS-CoV-2 spike (S) and nucleocapsid protein (NP) antigens	1 and 0.5 pg/mL	It provides superior fluorescence signals, enrichment capacity, and detectability for antigen tests	It is limited by the weak magnetic response, resulting in time consumption and sample waste in the magnetic separation process	[156]
Zinc oxide/reduced graphene oxide (bZnO/rGO) nanocomposite	Photochemical reduction	SARS-CoV-2 nucleocapsid (N) protein antigens in spiked	21 fg/mL over a linear range of 1–10,000 pg/mL	A nanocomposite coated on screen-printed carbon electrodes is used for the electrochemical immunobiodetection of capsid of SARS-CoV-2. The immunobiosensor provides a low detection limit over a wide linear range and exhibits adequate sensitivity for the detection of N protein in spiked samples	In some studies, graphene can have toxic effects on human and animal cells, especially when they are in the form of nanoparticles	[157]
Zinc sulfide/graphene hybrid	Microwave irradiation	Nucleic acid SARS-CoV-2	3.5 × 10^−15^ M	It provides a much higher current due to the presence of a large amount of conductive graphene, indicating a slow kinetic electron transfer process. Detects low concentrations of all different SARS-CoV-2 samples, using S, ORF 1a, and ORF 1b gene sequences as targets	No particular trend was found for these samples; the results can be inconsistent	[158]
CdSe@ZnS−COOH quantum dots (QDs)	Reduction and colloidal method	SARS-CoV-2, influenza A virus, and human adenovirus	8 pg/mL, 488 copies/mL, and 471 copies/mL	They provide larger reaction interfaces and specific active surface areas, higher QD loadings, and better luminescence and dispersibility than traditional spherical fluorescent microspheres for LFA applications	Only a test sample was used. It is necessary to conduct more tests since it is a good candidate, and experiments should be carried and its effectiveness should be corroborated external factors	[159]
Functionalized Co_2_ FeAl Nanoparticles	Coprecipitation method	RT-LAMP assay for the accurate detection of SARS-CoV-2 virus	10 copies of SARS-CoV-2 virus	Completely eliminated the incidence of false positives since it has good magnetic separation (because it uses a novel nanocapture system)	More tests need to be conducted to confirm that the false positives were eliminated	[160]
DNA-Functionalized Ti_3_C_2_ Tx MXenes	Noncovalent adsorption (soft epitaxy)	SARS-CoV-2 Nucleocapsid Gene	10^5^ copies/mL	Sensitive and selective detection of the SARS-CoV-2 N gene using nucleic acid hybridization and chemoresistive transduction	It has some drawbacks such as a long processing time, tedious sample preparation, and the need for laboratory facilities, which limits its applicability	[161]

**Table 2:** Advantages and disadvantages of hierarchical nanostructures, as well as their LOD for the detection of SARS-CoV-2, focusing on the biosensing method.

Nanostructure(s)	Method of Measurement	Analyte(s)	Detection Limit	Advantages	Disadvantages	Reference
AuNPs assembled by means of reduced graphene oxide (rGO) nanosheets	SEM	N protein SARS-CoV-2	13 fm	It can measure the change in dielectric properties of an electrode surface as a result of antibody–protein interactions. Detection is achieved in seconds and the sensor displays an excellent LOD and optimal detection range. Additionally, the sensor can be regenerated at least 10 times, reducing the cost per test	They can only be detected after 8 days of infection and if their concentration remains constant for a long period of time	[170]
ACE2-functionalized gold nanoparticles (AuNPs)	SERS, SEM	Angiotensin-converting enzyme 2 (ACE2)	80 copies mL^−1^	The SERS sensor functionalized with ACE2 is capable of accurately capturing unknown coronaviruses as long as its S protein can combine with the ACE2 protein	There are too many other proteins and biomacromolecules in the water contaminated by the SARS-CoV-2 virus, so these optically engineered SERS substrates suffer from overwhelming Raman signals from other impurities, leading to a poor signal-to-interference ratio	[171]
The ITO substrate modified with AuNPs@rPGO	NIR laser, SERS, and electrochemical techniques	SARS-CoV-2 spike protein	39.5 fmol L^−1^	Both Raman signals and electrochemical conductivity are improved based on both AuNPs and the graphene material. As a result, an excellent ability to monitor a wide range of COVID-19 protein concentrations was observed	Other sensors show better sensitivity. Concentrations of 50 nmol L^−1^ were used to see the results of the SERS intensities and only those quantities were used. It would be good if it could handle different saturations to observe the effectiveness and thus be able to ensure that it is a reliable test at the point of care	[172]
Synthesized CoFeBDCNH_2_-CoFe_2_O_4_ MOF-nanohybrid-modified gold chip	X-ray, PXRD, FETEM, FESEM, XPS, IR	SARS-CoV-2 spike glycoprotein	6.68 fg/mL and 6.20 fg/mL	The synergistic effects of CoFeBDCNH_2_-MOF and CoFe_2_O_4_ nanomaterials as MOF nanohybrids led to improvement in electrochemical detection of various targets, which can be used not only to detect the SARS-CoV-2 viral antigen but also any other disease-based biomarker	It is proposed to perform this test in real and time-dependent samples, including human saliva or nasal swabs since it is necessary to check if it is effective in these biological samples	[173]
Products based on DNA circuit and g-CNQDs@Zn-MOF	Fluorescence spectroscopy	SARS-CoV-2 nucleocapsid protein	1.0 pg/mL	It significantly amplified the signals while maintaining a lower background, so the fluorescence emission spectra were higher. Amplifying the signals allowed for a sensitive and highly specific detection of SARS-CoV-2	Long hours of analytical time and high price make it difficult to implement around the world	[174]
Multilayer silica-QD nanobead with Au	Fluorescence spectroscopy	FluA and SARS-CoV-2	5 pg/mL and 50 pfu/mL	QDs generate high luminescence and surface carboxyls are suitable for surface functionalization and generating hydrophobicity and colloidal stability. This can results in a high performance in biological samples in terms of sensitivity, stability, specificity, and reproducibility	SiO_2_ alone does not show any obvious fluorescent signal. These defects make it difficult to implement it in the diagnosis of respiratory viruses	[175]
Fe_3_ O_4_/Au/AgNPs	HAADF-STEM	SARS-CoV-2 RNA	6.1 ng/mL	An autonomous movement generates stronger mass transfer and therefore, a higher probability of capture and hybridization of the target viral RNA	Strains with a single mismatched base showed an obvious decrease in signal compared to perfectly matched sequences due to reduced hybridization efficiency. Non-complementary sequences are not detected in the assay because they do not react with the ssDNA probes and are left unhybridized	[176]
Nanohybrid Au@Ti_3_C_2_	SEM, TEM, FT-IR, EIS, CV	RNA-dependent RNA polymerase gene SARS-CoV-2	0.21 fM	Possible luminescence mechanism of the ECL biosensor makes a sensor with a wide detection range and a low detection limit	In this system, the ECL biosensor could not perform the “shutdown signal” state	[177]
Ag–Au NP alloy film	Optical spectrometer	SARS-CoV-2 spike glycoprotein (SARS-CoV-2 S2)	26.8 pM	Better light intensity in the two light paths of the interference structure resulted in a higher contrast ratio of the transmission spectrum and a more uniform distribution of interference peaks	The HCF simply acts as a beam splitter in the hollow core area and the effective detection area is the core surface of the HECF, resulting in a slight change in the power and contrast ratio of the sensor’s transmission spectrum	[178]
Antibody-functionalized silver microplasma-engineered nanoassemblies (AgMENs)	SERS, TEM SEM, XRD, XPS	SARS-CoV-2 nucleocapsid protein and spike protein variants	1 fg mL^−1^ and 0.1 pg mL^−1^	They greatly improve the molecular adsorption for biomolecular detection. They have a high adsorption capacity, and the coupling of the electromagnetic field into the porous structures improved the SERS response, allowing for the detection of target antigens even at low concentrations	The levels of immunoglobulins, such as IgM and IgG, can only be detected approximately 10 to 14 days after infection	[179]
Ti_3_C_2_ nanosheets and PDA–Ag nanoparticles	UV–Vis	SARS-CoV-2 S1 spike protein	12 fg mL^−1^	The analysis showed good reproducibility and high specificity, a wide linear range, and low LOD. It can be reprogrammed to detect any protein antigen if the corresponding specific nanobody is available	The study used artificial saliva and human serum but did not use complex body fluids	[180]
TiO_2_ @Bi_2_ WO_6_ y Ag_2_ S hollow microspheres	HRTEM, SEM, EDS, XRD, EIS	SARS-CoV-2 nucleocapsid protein	0.38 pg/mL	The system, TiO_2_ and Bi_2_ WO_6_, formed heterojunctions, which improved the absorption of visible light by using Ag_2_S. The hollow microspheres were sensitized, which effectively improved the photocurrent response, resulting in high sensitivity and good selectivity, reproducibility, and stability	TiO_2_ has a wide energy gap, so it could only absorb ultraviolet light from solar energy, which results in an inefficient use of light	[181]
Cu (OH)_2_ nanorod arrays	X-ray, XRD, and FESEM	SARS-CoV-2 spike glycoprotein	0.03 fg mL^−1^	Cu (OH) 2NRs, by providing a highly active surface, can not only act as a biocompatible scaffold to anchor aptamer chains and charge them further, but also as an electrochemical probe, which resulted in a wide dynamic range, high sensitivity, excellent sensitivity, low cost, good stability, good accuracy, and a low detection limit	The concentration and time required for aptamer immobilization were optimized as important factors in the preparation of aptasensors. Therefore, the concentration of aptamers on the electrode surface could directly affect the capture efficiency of the target	[182]
Nanohybrid MIP–aptasensor based on Ni_3_(BTC)_2_ MOF	FESEM, EDS	SARS-CoV-2 S protein-specific aminoaptamer	3.3 ± 0.04 PFU/mL	The high biocompatible surface increases the charge of the aptamer by covalent bonds, as well as the presence of cavities, which would increase the sensitivity of the electrochemical measurements	Aptamer performance may be affected by acidic solvents and some nuclease enzymes at high temperatures	[183]
Silicon nanoparticles and SiC@RP composite semiconductor SERS substrate	SERS and SEM	SARS-CoV-2 in saliva	7.6 × 10^−11^ g/mL	SiC maintains promising biocompatibility, stability, and electrical properties. A significant SERS effect was found, which improved charge transfer	SiC can only offer a weak SERS enhancement factor at a low level, which is a bottleneck found in most semiconductor substrates	[184]
SnS_2_ with “nano-gun” hierarchical nanostructure	XPS, SEM, TEM, and HRTEM	SARS-CoV-2 S protein and RNA	10 PFU/mL, 18 copies/mL, and 10^−13^ M	A unique hierarchical nanogun structure of SnS microspheres was produced for capillary effect-triggered molecular enrichment. Furthermore, it benefited from the contribution of lattice tension and sulfur vacancies for chemical enhancement. SnS_2_ microspheres exhibited an ultra-low LOD	When the concentration of MeB was less than 10^−10^ M, the Raman intensity no longer decreased linearly with decreasing concentration of MeB molecules. The viral culture method suffers from the disadvantages of a long culture time and complicated experimental operation	[185]

**Table 3:** Advantages and disadvantages of the hierarchical heterostructures for biosensing, as well as their LOD for the detection of SARS-CoV-2, focusing on the morphology of the building blocks of the nanostructures.

Nanoparticle(s)	Type of Nanostructure	Analyte(s)	Distribution of Building Blocks	LOD	Advantages	Disadvantages	Reference
MB-CD nanodots and AuN	Spherical and triangular gold nanoparticles	SARS-CoV-2	AFM images showed average length and height	2.0 aM	The developed DNA sensor is capable of detecting a SARS-CoV-2 sequence with a detection limit of 2.0 aM and in the presence of other possible interfering sequences corresponding to other viruses	Fluorescence was only observed for modified electrodes	[205]
Magnetic molybdenum trioxide @gold	Nanospheres	Spike protein of SARS-CoV-2	SEM images showed well-defined hierarchical nanostructures with average diameters	~4.5 fg mL^−1^, ~9.7 fg mL^−1^	The proposed MINERS-based biosensor not only provides exceptionally and stably enhanced Raman signals in a magnetic field, but also exhibits excellent repeatability and point-to-point consistency in the produced signals	The result is the formation of “hot spots”. It is at odds with the experimental results and the understanding of the real mechanisms of the MINERS approach since it requires advanced knowledge of quantum mechanical and plasmonic calculations	[206]
Au nanoparticles and TiO_2_	Nanoparticle spheres	SARS-CoV-2 spike protein	In the SEM images, stacked spheres and nanoparticles with different sizes were observed	5 fg mL^−1^	The SPR effect of Au nanoparticles can greatly enhance the nanomaterial with stronger visible light harvesting, faster transmission efficiency of photoinduced charges, and better photoelectric signals than TiO_2_. This immunosensor achieved satisfactory sensitivity, stability, reproducibility, and applicability	When AA concentrations are higher than 0.1 mol L^−1^, the photocurrent response of SP/BSA/Nb/Au@TiO_2_/ITO decreases slightly because AA can retard the electron transfer between them and the surface of the electrode	[207]
Gold nanoparticles	Spherical Au NPs	Spike protein of SARS-CoV-2	The Au NPs were monodisperse and not aggregated	1 pg/mL	The assay developed with gold enhancement was able to detect inactivated SARS-CoV-2 virions at 500-fold lower dilutions compared to the conventional assay. This approach uses stable reagents, facilitates rapid (8 min) and easy point-of-need testing, and could potentially be used in practice outside the laboratory	Non-specific TZ staining was observed for this enhancement method	[208]
SiO_2_ @Au CSNPs	Gold nanoparticle-assembled SiO_2_ core-satellite	SARS-CoV-2 nucleocapsid protein	Showed numerous SiO_2_ @Au CSNPs undesirably trapped in small-sized pore membranes	0.24 pg mL^−1^	AuNP satellites in the synthesis of SiO_2_ @Au CSNP significantly improved its light absorption and detection sensitivity and lowered the detection limit by 2 orders of magnitude relative to conventional gold colloids. They also enabled highly sensitive detection of the SARS-CoV-2 nucleocapsid protein	In different studies, the main drawback of silica is that it has a high melting point, which means that it requires more energy to melt than many other semiconductors. This needs to be taken into account for mass production	[209]
AuNP, rGO, PMB/PILs, and PILs	Nanosheets	SARS-CoV-2 spike protein	The SEM images showed that AN exhibits a very polished surface, while on the surface of the AuNP/AN, well-distributed AuNPs with an average size were observed	0.1~1000 ng mL^−1^	The developed miniature biosensor exhibits high specificity and stability	The maximum currents decrease noticeably at some points due to the poor conductivity of the SARS-CoV-2 protein	[210]
ASO/AuNPs	A box shape is shown	N gene of the SARS-CoV-2 viral genome	TEM images showed that ASO-capped AuNPs were individually dispersed without visible aggregation	0.18 ng/μL	The current methodology guarantees its viability even with mutated N gene forms of the virus during its spread, since the assay has been designed to simultaneously target two separate regions of the gene	An increase in absorbance was observed at a wavelength of 660 nm with a redshift of ~40 nm with a color difference from violet to dark blue, but a marked change in visual appearance was desired if it is to be used for the detection of SARS-CoV-2	[211]
Fe_3_ O_4_–Au nanoparticles	Nanoneedle array and microsphere microstructure	SARS-CoV-2 from nasal and throat swabs	The HRTEM image showed that Fe_3_O_4_ was made up of many ultrafine nanoparticles	100 copies/mL	The optimal magnetic SERS biosensor with high sensitivity was obtained	The Raman intensity was proportional to the amount of analyte, so the 5- to 6-fold gradual growth of the Fe_3_O_4_-Au nanocomposite was the best magnetic SERS substrate for SERS detection. However, compared with 5-fold grown Au NPs, 6-fold growth would cause severe agglomeration and be more time-consuming	[212]
Fe_3_ O_4_	Spherical shape	SARS-CoV-2 N protein	TEM images revealed that Fe_3_ O_4_ presented a uniform spherical shape with an average diameter of approximately 180 nm	2.9 pg mL^−1^	It had an excellent magnetic response that can simplify the separation and washing process, while allowing immunological recognition to be carried out in the liquid phase. The PEC immunosensor exhibited a wide linear range and low detection limit, providing an applicable method for the diagnosis of SARS-CoV-2	At one point in the experiment, the union of these nanoparticles resulted in the accumulation of electrons in the CB of TiO_2_ and holes in the VB of CdS. Therefore, a good method must be sought so that the generated electrons are easily transferred	[213]
Ti_3_ AlC _2_MXene/P–BiOCl/Ru(bpy)_3_ ^2+^	2D sheet-shaped nanostructure with stacked distribution	SARS-CoVNP	SEM showed that Ti_3_ AlC _2_ has a highly stacked morphology and showed that MXene after etching and exfoliation has a larger interlayer spacing compared to Ti_3_ AlC_2_	0.49 fg/mL (/N = 3)	Performs reliable recognition of CoVNP, obtaining a wide linear range and low LOD. Furthermore, combining 2D MXene architectures with 2D semiconductor materials has the potential for building high-performance ECL sensing platforms. Presents a new perspective for ECL applications in the field of nanomedicine in the prevention of various diseases	When MXene/P–BiOCl/Ru(bpy)_3_^2+^/GCE is sequentially anchored with Ab (curve d), BSA (curve e), and CoVNP (curve f), the ECL signal gradually decreases	[214]
ITO/GNPs@MUA	Sandwich-type and spherical nanoparticles	Spike protein RBD	The SEM images proved that GNPs present as uniformly dispersed spheres and numerous groups of GNPs@MUA	0.577 fg mL^−1^	The sandwich immunosensor showed high sensitivity, a low detection limit, good repeatability, and perfect reproducibility	Although the sensor shows good results in its sensitivity, it is necessary to apply it not only to artificial saliva, but also to human saliva samples, to corroborate the data.	[215]
Monolayer Ag nanoparticles (MAgNPs) covered with graphene	Nanosheet nanostructure	SARS-CoV-2 spike protein	They have a uniformly rough surface	0.1 fg mL^−1^ and 10 fg mL^−1^	In comparison with polymerase chain reaction (PCR), surface-enhanced Raman scattering (SERS) is a promising method for detecting SARS-CoV-2 due to its fast, easy operation, and high-sensitivity properties	A disadvantage in the study would be its preparation method since in some studies, CVD tends to form tensions, higher diffusion rates of the elements from the substrate to the film, and the possible degradation of the substrate	[216]
Ni (OH) _2_ NP @ SPCE of carbon	Flower-shaped nanostructure	Ultrasensitive detection of antibodies against SARS-CoV-2	FESEM images showed that the surface of SPCE consists of a uniform layer of carbon particles	1 fg mL^−1^ to 1 µg mL^−1^	The electrochemical biodevice was able to accurately and easily detect and determine IgM/IgG levels in human blood serum with a remarkable detection limit in less than 20 min with a wide dynamic range	The biosensor is sensitive to other types of coronaviruses, which would affect the search for a biosensor that only detects SARS-CoV-2	[217]
IPCF	Nanostructured pattern with nanoholes	Spike proteins in artificial saliva	SEM image showed that the IPCF had a nanostructured pattern with periodically arranged nanoholes	429 fg/mL	The IPCF sensor is expected to be suitable for widely available and highly usable antigen tests with smartphones and can be used in digital medical diagnostic systems, such as POC	When the sample concentration was 1 pg/mL, the same response was obtained between the PBS and saliva sample solutions. Contaminants adsorbed non-specifically to the saliva samples were expected to increase the response in the low peak concentration range	[218]
Pt/MoS_2_	Nanosheet	S1 protein and SARS-CoV-2 pseudovirus	TEM image showed uniform distribution of elements in the sample	0.53 ng/mL	The formation of 1 T-MoS_2_ within the Pt/MoS_2_ heterostructure improved the material’s conductivity, stability, light harvesting ability, and photoelectric conversion capacity. It could specifically capture the S1 protein, causing a decrease in photocurrent intensity due to high steric hindrance and low conductivity	It is possible that if there was an excessively high electrical pulse, it would cause photocurrent overflow under the same conditions, so these conditions must be prevented	[219]
TiO_2_ (Co-TNT)	Nanotubes	SARS-CoV-2 S-RBD protein	The SEM images showed the presence of precipitates on the TNT surface	0.7 nM	This simple, inexpensive, rapid and non-invasive diagnostic platform has the potential to detect SARS-CoV-2 in clinical samples, including nasal, nasopharyngeal swab, and saliva samples	The molecular weight of the S-RBD protein was slightly larger (~35 kda) compared to the calculated size	[220]
MOF-5/CoNi_2_ S_4_	Semi-cylindrical nanostructures	SARS-CoV-2 recombinant spike antigen	AFM results showed different types of roughness at different points. At other points, the roughness was more homogeneous than the other two points mentioned	5 nM	The use of the inorganic–organic nanocomposite composed of MOF-5 and porphyrins could reduce interactions with cell walls	The presence of CoNi_2_ S_4_ increased cytotoxicity due to nickel, which is highly toxic even at ppm concentrations	[221]
SiNWs/AgNPs	Nanowires	SARS-CoV-2 (S protein)	A highly ordered vertical nanowire arrays with homogeneous decoration using spherical AgNPs	0.90 µm	The SiNW/AgNP sensor platform was sensitive and accurate for the SARS-CoV-2 spike protein even at an exceptionally minimal picomolar concentration	This study was a preliminary study and the authors aim to develop a direct detection method for SARS-CoV-2 virus at the protein level (spike RBD) and needs further improvements (labeled detection for specific targets) to promote it as a real-time monitoring diagnostic device	[222]
Graphene-based	Quadratic epitaxial heterostructure	SARS-CoV-2 spike protein S1 antigen	AFM images indicate indicating the root-mean-square roughness	60 copies/mL, 1 ag/mL	The sensor is reusable, allowing for reimmobilization of the crosslinker and antibodies on the biosensor after desorption of biomarkers by NaCl solution or heat treatment above 40 °C	The resulting bias field of the PLL is reduced. It produces inhomogeneity in the field, which can generate stress in the underlying graphene sheet. This inhomogeneity would produce a compressive stress as it removes the positive charge that interacts with the positively charged QFS EG layer. Therefore, this stress would reduce the local carrier concentration and induce stress on other nearby C-C bonds, propagating its effect	[223]

## Abbreviations

ACE2	Angiotensin-converting enzyme 2
AFM	Atomic force microscopy
BPV	Bioinspired plasmovirus
CdS	Cadmium sulfide nanoparticles
cDNA	Copy DNA
COVID-19	Coronavirus disease 19
CoVNP	Nucleocapsid
CRISPR	Family of DNA sequences found in the genomes of prokaryotic organisms such as bacteria
CS	Chitosan
DNA	Deoxyribonucleic acid
DPV	Differential pulse voltammetry
CV	Cyclic voltammetry
ECL	Electrochemiluminescence
EDL	Electrical double layer
EDS	Energy dispersive spectroscopy
EDX	X-ray spectroscopy
EIS	Electrochemical impedance spectroscopy
FESEM	Field-emission scanning electron microscopy
FE-TEM	Field-emission transmission electron microscopy
FluA	Influenza A virus
FTIR, FT-IR or IR	Fourier transform infrared spectroscopy
GO	Graphene oxide
GO/AuNPs	Graphene/gold nanoparticle
GOD	Glucose oxidase
GSH	Glutathione
HAADF-STEM	High-angle annular dark-field scanning transmission electron microscopy
HCF	Hollow core fiber
HECF	Hollow eccentric core fiber
HRP	Detection based on biotinylated molecules
HRTEM	High-resolution transmission electron microscopy
ICA	Immunochromatographic assay
IPCF	Imprinted photonic crystal film
ITO	Indium tin oxide
LFA or LFIAs	Lateral flow immunoassay
LOD	Limit of detection
LSPR	Localized surface plasmon resonance
MB-CD	Methylene blue functionalized carbon
MINERS	Magnetically-induced nanogap-enhanced Raman scattering
MIP	Molecular imprinting polymer
MNPs	Magnetic nanoparticles
MOF or MOFs	Metal–organic frameworks
MS	Mass spectrometry
MUA	Mercaptoundecanoic acid
MWCNT	Multi-walled carbon nanotubes
N	Nucleocapsid
NGs	Nanoghosts
NIR	Near-infrared
NP	Nucleocapsid protein
NPs	Nanoparticles
NW	Nanowires
ORF	Open reading frame
PANi	Hybrid polyaniline
pCRISPR	Plasmid for reconstituting the CRISPR system
PDA	Polydopamine
PDMS	Polydimethylsiloxane
PEC	Photoelectrochemical
PEI	Polyethyleneimine
PGE	Pencil graphite electrode
PILs	Poly(ionic liquids)
PL	Photoluminescence spectroscopy
PMB/PILs	Provides π-π interactions with poly(methylene blue)
PNA	Peptide nucleic acid
POC	Point-of-care
PPT	Plasmonic photothermal
PPy	Platform made of polypyrrole
PVDF	Polyvinylidene fluoride
QD or QDs	Quantum dots
rGO	Reduced graphene oxide
RdRp	RNA-dependent RNA polymerase
rPGO	Reduced porous graphene oxide
RT-LAMP	Reverse transcription loop-mediated isothermal amplification
RT-PCR	Reverse transcription polymerase chain reaction
S	Spike
SAM	Self-assembled monolayer
SARS	Severe acute respiratory syndrome
SARS-CoV-2	Severe acute respiratory syndrome coronavirus 2
SM	Saturation magnetization
ssDNA	Single-stranded DNA
SEM	Scanning electron microscopy
SERS	Surface-enhanced Raman spectroscopy
SM	Saturation magnetization
SPCE	Screen-printed carbon electrode
SPR	Surface plasmon resonance
TEM	Transmission electron microscopy
TZ	Test zone
UV-Vis	Ultraviolet (UV) spectroscopy
X(XRD)	X-ray diffraction
X(XPS)	X-ray photoelectron spectroscopy
ZnO/rGO	Zinc oxide/reduced graphene oxide
